# Severe COVID-19 in pregnancy is almost exclusively limited to unvaccinated women – time for policies to change

**DOI:** 10.1016/j.lanepe.2022.100313

**Published:** 2022-01-26

**Authors:** Hilde Engjom, Thomas van den Akker, Anna Aabakke, Outi Ayras, Kitty Bloemenkamp, Serena Donati, Danilo Cereda, Evelien Overtoom, Marian Knight

**Affiliations:** a Dept of Mental and Physical Health, Norwegian Institute of Public Health, Bergen, Norway; b Department of obstetrics and gynaecology, Leiden University Medical Center, Leiden, the Netherlands; c Athena Institute, VU University, Amsterdam, the Netherlands; d Dept of Obstetrics and Gynecology, Hillerød Hospital, Nordsjaellands Hospital, Denmark; e Dept obstetrics and Gynecology, Copenhagen University Hospital, Holbæk, Denmark; f Dept of Obstetrics and Gynecology, Helsinki University Hospital, Helsinki, Finland; g Department of Obstetrics, WKZ Birth Centre, Division Woman and Baby, UMC Utrecht, Utrecht, the Netherlands; h National Centre for Disease Prevention and Health Promotion, Istituto Superiore di Sanità - Italian National Institute of Health, Rome Italy; i Regione Lombardia DG – Welfare, Milano, Italy; j National Perinatal Epidemiology Unit, Nuffield Department of Population Health, University of Oxford, Oxford, UK

Pregnant women continue to be excluded from most clinical trials of COVID-19 vaccines and medication, despite very clear pre-pandemic guidance.^1^ There appears little incentive amongst regulators or pharmaceutical companies to change this. Compounded by their exclusion, there is considerable vaccine hesitancy amongst pregnant women.^2^ Such hesitancy persists, even though at present adverse outcomes of SARS-CoV-2 infection are increasing among pregnant and postpartum women in many countries,^3^ while these are improving in most other groups. The impact of the omicron variant is, as yet, unknown.

Vaccine hesitancy in pregnancy is not a new phenomenon. Salmon and colleagues^4^ identified three factors which influence parents’ acceptance of vaccines for either them or their children: confidence in the efficacy of the vaccines, trust in their healthcare professionals, and, importantly, certainty of the systems to assess vaccine safety. They note that addressing vaccine hesitancy is a “complex problem [which] requires a multilevel approach, including interventions at the individual and health system levels.”

Data suggest that vaccines are a highly effective protection against severe COVID-19 in the non-pregnant populations in which they were initially tested.^5^ However, in many countries, pregnant and postpartum women and those planning a pregnancy continue to receive conflicting messages, mainly regarding the safety of the vaccines. Misleading information on social media continues to impede uptake of vaccination in pregnant and postpartum women, even though observational data about vaccine safety, now including more than 250 000 women, are very reassuring.^6^ Additionally, clear potential benefits have been documented, such as placental transmission of protective antibodies to the fetus.^7^ By preventing maternal disease, vaccination may prevent stillbirths, preterm births and associated neonatal deaths.

Simultaneously, it has become clear that pregnant and postpartum women are at higher risk of serious illness compared to their non-pregnant contemporaries. This seems especially true for the Delta variant, which increased the risk of intensive care unit admission among pregnant women 2-3 times, with a 50% increase in iatrogenic preterm births.^3^^,^^8^^,^^9^ Several European countries (Norway, UK) have recently recognised pregnant and postpartum women as an ‘at risk’ priority group for COVID-vaccination. This policy has been in place for even longer in other countries (Belgium, Denmark), yet it is still not universal (for example, in Italy, the Netherlands and Finland pregnant women are not prioritised).

Multiple initiatives to promote uptake of COVID-vaccination in pregnancy have been undertaken, with widely differing uptake rates and uptake estimates varying between 22% in England and 80% in Norway. Surprisingly, data are not available on Covid-19 vaccination rates amongst pregnant women in all European countries. Within the International Network of Obstetric Survey Systems (INOSS)^10^ we have been able to combine surveillance data in six countries showing that amongst the most critically ill pregnant and postpartum women, almost none were vaccinated (Table 1). This is observed despite widely varying population vaccine uptake rates.

**Table 1:** Admissions of symptomatic pregnant women to hospital and critical care with estimated vaccine uptake rates, six European countries, May-December 2021.

Country	Period covered	Number of women admitted to hospital with covid	Number admitted to critical care (% of those admitted to hospital)	Number admitted to critical care who are unvaccinated (% of those admitted to critical care)	Estimated proportion of pregnant population who have received at least one vaccine dose
**UK**	16/05/21-31/10/21	1436 (symptomatic only)	230 (16)	225 (98)	22% (England, August 2021) 43% (Scotland, October 21)
**Netherlands**	01/05/21-06/12/21	220 (symptomatic only)	52 (24)	47 (90) Unknown: 5 (10) Vaccinated: 0 (0)	30-50%
**Norway**	15/07/21-15/12/21	28^a^ (symptomatic only)	8 (29)	8 (100)	80%^b^
**Finland (Helsinki Region)**	01/07/21-15/12/21	11 a(symptomatic only)	5 (45)	5 (100)	60%^c^
**Denmark**	01/06/21-30/11/21	N/A	8 (N/A)	8 (100)	56% (November 2021)
**Italy (Lombardy Region)**	01/05/21-15/12/21	506^d^	15 (3)	12 (80) received one dose: 3 (20)	20 % (May-October 2021)

N/A – not available data

a Reporting of severe cases has been verified, there may be under-reporting of pregnant women with less severe disease in November and December 21.

b Estimated at the Norwegian Institute of Public Health, 15 December 21.

c Estimated by the Finnish Institute of Health and Welfare, 20 December 21.

d All pregnant women, symptomatic and asymptomatic.

Our findings emphasize the message to unvaccinated pregnant women, their partners, health professionals caring for pregnant women, decision makers and politicians that vaccination protects against severe disease. As the world is entering a new phase of the COVID-pandemic, with the Delta-variant rapidly being overtaken by the Omicron-variant, booster vaccinations are increasingly important to provide protection against severe COVID-19, especially in vulnerable groups such as pregnant and postpartum women or women who want to become pregnant. However, large numbers of pregnant and postpartum women in low, middle and high-income settings have yet to receive even a single vaccination dose. Health system as well as individual actions are now clearly needed. The World Health Organisation recommends COVID-19 vaccination in pregnant women when the benefits of vaccination to the pregnant woman outweigh the potential risks. These multi-country data clearly show those benefits in terms of prevention of severe disease. We believe that all governments should now prioritise pregnant and postpartum women as an at-risk group and encourage their vaccination.

## Declaration of Competing Interest

Hilde Engjom reports funding from the Nordic federation of Societies of Obstetrics and Gynecology (NFOG) research fund and cost refund for travel costs as invited key note speaker at the Norwegian Perinatal Society annual meeting Nov 2021; Marian Knight reports funding from the National Institute or Health Research, the Medical Research Council and Wellbeing of Women; Thomas van den Akker reports funding from the Medical Research Council and the Laerdal Foundation; Anna Aabakke reports funding from The Region of Southern Denmark and Region Zealand's shared fund for joint health research projects and is head of the educational committee of the Danish Society of Obstetrics and Gynaecology (DSOG); Outi Ayras reports funding from the Finnish Medical Foundation and the Nordic Federation of Obstetrics and Gynaecology; Kitty Bloemenkamp reports frunding from the European Medicines Agency (EMA) ‘COVID-19 infectiOn aNd medicineS In pregnancy’ for the INOSS network; Serena Donati reports funding from the Istituto Superiore di Sanità (Italian National Health Institute). Danilo Cereda and Evelien Overtoom have no interests to declare.

## Appendix Supplementary materials

Image, application 1
