# Supplementary material for: Severe COVID-19 in pregnancy is almost exclusively limited to unvaccinated women – time for policies to change
Source: Lancet Reg Health Eur. 2022 Jan 26;13:100313. doi: 10.1016/j.lanepe.2022.100313 (PMC8791061; doi:10.1016/j.lanepe.2022.100313)
Supplement: Supplementary file 1 [file mmc1.docx]

**Supplementary material: Abstract translation to participating country languages**

*The following translations in Finnish, Danish, Italian, Dutch, and Norwegian were submitted by the authors and we reproduce them as supplied. They have not been peer reviewed. Our editorial processes have only been applied to the original abstract in English, which should serve as reference for this manuscript.*

Table of contents

[Finland 1](#_Toc93479079)

[Denmark 1](#_Toc93479080)

[Italy 1](#_Toc93479081)

[Netherlands 2](#_Toc93479082)

[Norway 2](#_Toc93479083)

Finland

Raskautta suunnittelevat, raskaana olevat ja vastikään synnyttäneet kokevat epäluottamusta koronavirusrokotteita kohtaan. Osasyynä tähän on rokotteita koskevien ohjeiden ja suositusten muuttuminen tutkimustiedon karttuessa.

Kansainvälinen INOSS (the International Network of Obstetric Survey Systems) tutkimusryhmä keräsi tietoa koronaviruksen delta-variantin aikaisista tehohoidetuista raskaana olleista tai vastikään synnyttäneistä äideistä. Valtaosa näistä potilaista oli rokottamattomia. Rokotesuositukset ja rokotekattavuus raskaana olevilla vaihteli suuresti maiden välillä.

Raskaana olevat tulisi luokitella koronavirusrokotusten suhteen priorisoitavaksi riskiryhmäksi ja kaikkien terveydenhuollon ammattilaisten tulisi suositella koronavirusrokotetta raskaana oleville.

Denmark

Informationen omkring vaccination af gravide og barslende mod SARS-CoV-2 var modstridende i pandemiens begyndelse, og vaccinetilslutningen I denne befolkningsgruppe er lav.

Forekomsten af svære tilfælde af SARS-CoV-2 blandt gravide og barslende kvinder smittet med delta-varianten er fundet høj i lande med populationsbaseret overvågning af SARS-CoV-2 smitte blandt gravide i regi af the International Network of Obstetric Survey Systems (INOSS). Derudover er størstedelen af de gravide og barslende, som indlægges på intensiv afdeling med SARS-CoV-2, uvaccinerede. Der er store forskelle i vaccineanbefalingerne og vaccinationsraten blandt gravide landende imellem, men disse data viser en tydelig beskyttende effekt af vaccine mod udvikling af svær COVID-19 sygdom.

Alle sundhedsfaglige personer bør opfordre til vaccination af gravide og vi mener, at vaccination af gravide og barslende skal prioriteres.

Italy

All’inizio della pandemia da SARS-CoV-2, le donne in gravidanza e puerperio e quelle che programmavano una gravidanza hanno ricevuto messaggi contrastanti circa l’opportunità di vaccinarsi e, ancora oggi, l’esitazione vaccinale in questa popolazione è forte.

Durante il periodo della variante Delta, i Paesi dotati di sistemi di sorveglianza attiva su base di popolazione che partecipano all'International Network of Obstetric Survey Systems (INOSS) hanno rilevato un aumento dei casi gravi di malattia da SARS-CoV-2 in gravidanza e puerperio e hanno dimostrato che la maggioranza delle donne ricoverate in terapia intensiva non era vaccinata. Nonostante i Paesi aderenti al network INOSS abbiano adottato politiche non uniformi circa i criteri di priorità per l’offerta vaccinale e presentino tassi variabili di accettazione del vaccino in gravidanza, i dati sugli esiti materni dimostrano chiaramente come, nel mondo reale, la vaccinazione protegga dalle forme gravi della malattia da COVID-19.

I professionisti sanitari dovrebbero pertanto promuovere la vaccinazione e i governi dovrebbero dare priorità all’offerta vaccinale in gravidanza e puerperio.

Netherlands Zwangeren, kraamvrouwen, en vrouwen die een zwangerschap plannen, hebben eerder in de COVID-pandemie tegenstrijdige berichten ontvangen over belang en veiligheid van vaccinatie. Deze berichten hebben bijgedragen aan het feit dat veel vrouwen zich nu niet laten vaccineren.

Landen met actieve populatie-brede registratiesystemen, verenigd in het International Network of Obstetric Survey Systems (INOSS), rapporteren hogere getallen van ernstige COVID onder zwangeren en kraamvrouwen met de delta-variant van SARS-CoV-2. Ook blijkt dat de grote meerderheid van zwangeren en kraamvrouwen op de intensive care niet gevaccineerd is. Er is geen eenduidig beleid tussen landen omtrent prioritering van vaccinatie onder zwangeren of andere manieren om de vaccinatiegraad te verhogen. Toch tonen de data duidelijk aan dat vaccinatie beschermt tegen ernstige ziekte.

Alle hulpverleners zouden vaccinatie moeten aanmoedigen, en regeringen zouden zwangeren en kraamvrouwen met prioriteit toegang moeten geven tot vaccinatie.

Norway

Gravide, barselskvinner og kvinner som planla å bli gravide fikk motstridende råd om vaksinasjon tidlig i SARS-CoV-2 pandemien og mange var nølende til å ta vaksiner.

Land med pågående befolkningsbasert overvåkning i samarbeidet International Network of Obstetric Survey Systems (INOSS) rapporterer om høyere antall alvorlige infeksjoner blant gravide og barselskvinner med delta varianten av SARS-CoV-2, og viser at flertallet av de gravide som ble innlagt for intensivbehandling var uvaksinerte. Både helsepolitiske retningslinjer knyttet til prioritering og gjennomføring av vaksinasjon og andelen gravide som er blitt vaksinert viser stor variasjon mellom landene, samtidig som overvåkningen klart viser at vaksinasjon beskytter gravide og barselskvinner mot alvorlig sykdom.

Helsepersonell bør tilråde vaksinasjon og helsemyndighetene må prioritere vaksine mot SARS-CoV-2 for gravide og barselskvinner.
